# Histology and ultrastructure of the integumental chromatophores in tokay gecko (*Gekko gecko*) (Linnaeus, 1758) skin

**DOI:** 10.1007/s00435-017-0348-9

**Published:** 2017-03-17

**Authors:** Paweł Szydłowski, Jan Paweł Madej, Marta Mazurkiewicz-Kania

**Affiliations:** 1 Department of Immunology, Pathophysiology and Veterinary Preventive Medicine, Faculty of Veterinary Medicine, Wrocław University of Environmental and Life Sciences, Norwida 31, Wrocław, 50-375 Poland; 2 Department of Histology and Embryology, Faculty of Veterinary Medicine, Wrocław University of Environmental and Life Sciences, Norwida 25, Wrocław, 50-375 Poland; 3 Laboratory of Microscopic Techniques, Faculty of Biological Sciences, The University of Wrocław, Sienkiewicza 21, Wrocław, 50-335 Poland

**Keywords:** Skin, Chromatophores, Melanophores, Pigment cells, *Gekko gecko*

## Abstract

This paper describes the relationship between the arrangement of dermal chromatophores in tokay gecko (*Gekko gecko*) skin and the formation of wild-type colouration, with emphasis on the ultrastructure of chromatophores. The samples of the tokay gecko skin were collected from wild-type colouration adult specimens. Morphology and distribution of chromatophores was determined by using light microscopy and transmission electron microscopy. The present study revealed that orange/red coloured skin of *G. gecko* contained erythrophores, which were located under basement membrane, and usually comprised deeper situated iridophores and melanophores which were form single layer with iridophores or were occupying the deepest region of dermis. In orange/red coloured skin, erythrophores were the predominant chromatophores. However in blue areas these cells occurred in small numbers or were not noticed at all. In blue pigmented areas predominated iridophores and melanophores. Iridophores were found just under basement membrane, but this superficial location of iridophores occured only in areas without erythrophores. Distribution of erythrophores, melanophores, and iridophores determines the characteristic blue colour of the tokay gecko skin with orange/red dots on the whole body.

## Introduction

The tokay gecko (*Gekko gecko*, Linnaeus, 1758) is a nocturnal species naturally occurring in Southeast Asia (Northeastern India, Birma, Anam, the Indian Archipelago) (Boulenger 1885) and introduced to USA (Means 1996). Wild-type adult tokay gecko has a skin pigmentation pattern with a blue colour background on the dorsal and ventral side of the head, body, and tail with orange or red dots.

Chromatophores that involve reptile skin colouration include epidermal melanocytes and dermal melanophores, xanthophores, erythrophores, and iridophores (Alibardi 2013; Saenko et al. 2013). Melanocytes and melanophores are dark cells which have characteristic melanin-containing vesicles called melanosomes. Xanthophores are yellow pigment cells containing vesicles filled with carotenoid or pterinosomes filled with pteridines (Bagnara 1966). Iridophores are light-reflecting cells that contain light-reflecting platelets made up of crystalline guanine inclusions (Kuriyama et al. 2006; Saenko et al. 2013). Erythrophores containing red pigments and which occurrence result in red skin colouration are described in *Phelsuma sp*. and *Furcifer pardalis* (Saenko et al. 2013; Teyssier et al. 2015). The precise thickness, spacing, and organisation of reflective platelets determine the ability of iridophores to reflect specific wavelengths (Huxley 1968; Denton and Land 1971; Teyssier et al. 2015). In some species of amphibians and reptiles, chromatophores form a dermal chromatophore unit. In this structure, processes of melanophores extend upward covering the more superficially located iridophores and xanthophores (Bagnara et al. 1968; Bagnara 1983; Taylor and Hadley 1970; Taylor and Bagnara 1972). In this system, xanthophores are associated with orange-yellow to red colour, while the light-reflecting iridophores generate a blue colour. Green pigmentation of skin in reptiles can emerge when yellow xanthophores are associated with blue iridophores (Bagnara et al. 2007; Saenko et al. 2013). Studies on the *Sphenodon punctaus, Plestiodon latiscutatus*, and *Phrynosoma modestum* have indicated that dermal melanophores contribute to the darkening of the skin in the form of stripes or dots either together with the external epidermal melanocytes (darkest colouration), or without the presence of the latter (Sherbrooke and Frost 1989; Kuriyama et al. 2006; Alibardi 2012). Recent studies have shown lack of iridophores in representative of nocturnal species—*Eublepharis macularius* (Szydłowski et al. 2016). In squamates another type of colour source, except occurrence of chromatophores and their structural or location relationships, was described, and depends on changes of pH or redox state of pigments (Saenko et al. 2013).

The distribution of chromatophores in skin of nocturnal species of reptiles is still not fully understood. The aim of this study was to examine the ultrastructure and arrangement of chromatophores in tokay gecko skin to explain how wild-type colouration is formed. According to previous studies on other squamata species our hypothesis predict prevalence of erythrophores in red coloured skin areas and prevalence of iridophores in blue coloured skin areas. To the best of the our knowledge, this is the second report of chromatophores in nocturnal species (the first in tokay gecko) and we hope to start a debate about the presence and function of iridophores in nocturnal species in Gekkota genera.

## Materials and methods

**Fig. 1:**
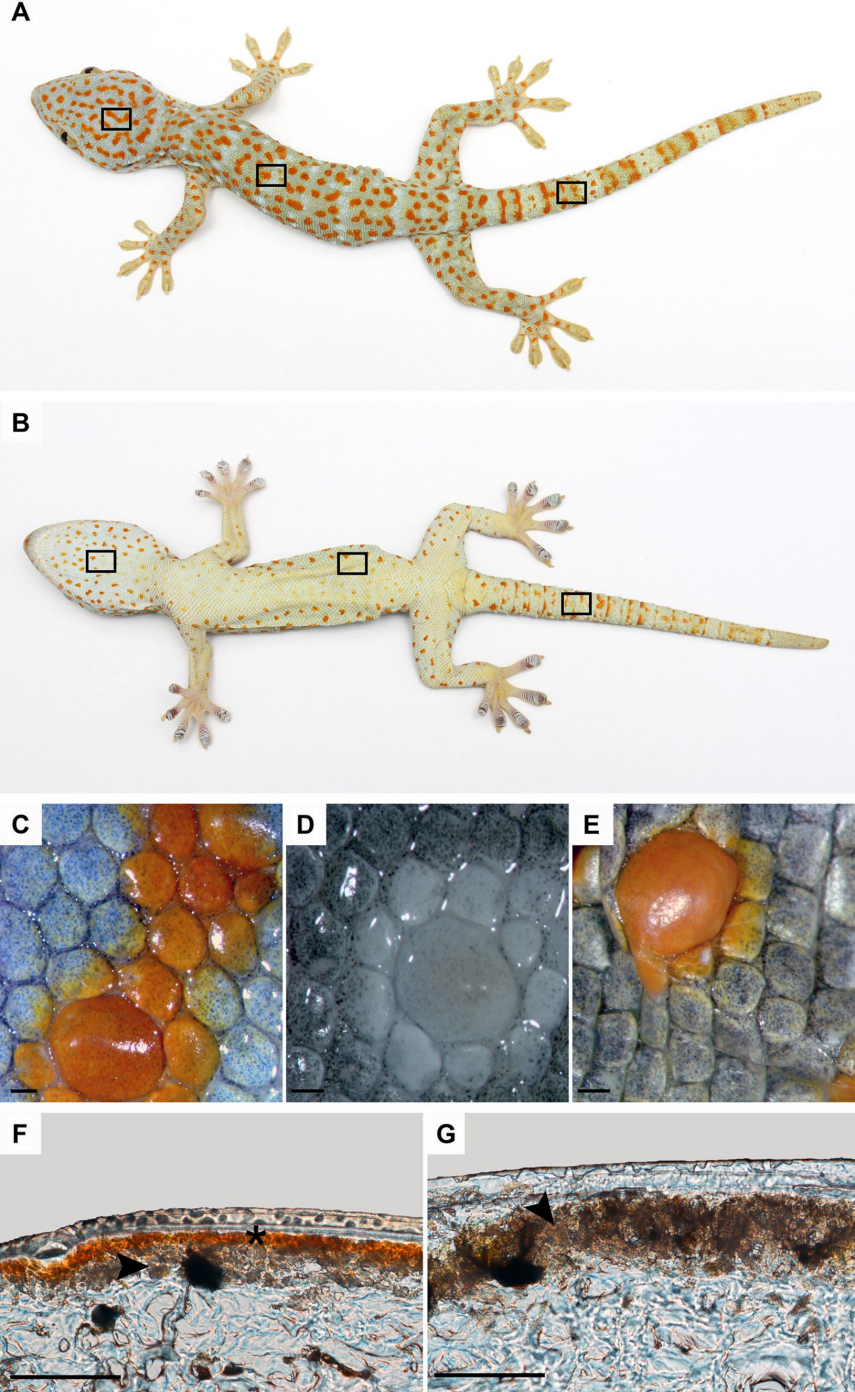
**A**, **B** Schematic drawing of tokay gecko skin regions where the samples were collected from. **C**–**E** View of unstained skin samples under stereomicroscope placed in Ringer's solution (**A**), 30% NH_4_OH (**B**) and 100% acetone (**C**); *scalebar* 1 mm. **F, G** Frozen cross sections of unstained skin samples; *scalebar* 50 µm. **F** Orange/red pigmented area from dorsal part of body with superficial layer of erythrophores (*black asterisk*) and deeper located iridophores (*black arrowhead*). **G** Blue pigmented area from dorsal part of body with visible iridophores (*black arrowhead*)

The material for the study was collected during necropsy of five already dead animals from private owners in the course of the routine pathological examination in Department of Epizootiology and Clinic of Bird and Exotic Animals (Wrocław University of Environmental and Life Sciences). Furthermore, the material was taken from 14 archived specimens from their natural habitats in Java Island and Indonesia, which were conserved in 70% ethanol at the Museum of Natural History of University of Wrocław, Poland. The samples of the tokay gecko skin were taken from wild-type colouration adult specimens and the cause of whose death had no influence on the skin condition. Samples of skin in blue and orange/red colouration were taken from the dorsal and ventral part of head, body, and tail (Fig. 1). We used to different microscopy techniques to distribution and ultrastructure of chromatophores. For light microscopy samples were fixed in 4% buffered formaldehyde or 70% ethanol and routinely processed in paraffin. Sections (7 µm thick; HM310, Microm, Walldorf, Germany) of each tissue were stained with Delafield’s hematoxylin (Roth GmbH, Karlsruhe, Germany) and eosin (Poch S.A., Gliwice, Poland)—H&E, and with Mallory trichrome stain. One portion of tissue was examined *in toto* in transmitted light to estimate the number and morphology of melanophores. Unstained skin samples were placed in Ringer’s solution to prepare 10 µm thick sections on cryostat (Leica CM1850, Leica Microsystems GmbH, Wetzlar, Germany). The slices were examined and photographed under a Nikon Eclipse 80i (Nikon, Melville, NY, USA) light microscope equipped with a video camera and with differential interference contrast (DIC, Nomarski contrast) to confirm the existence of iridophores. Sections for transmission electron microscope of each skin sample were fixed in 2.5% glutaraldehyde, postfixed in mixture containing 1% osmium tetroxide and 0.8% potassium ferrocyanide, dehydrated in acetone, and embedded in Epon. Epon blocks were cut on Reichert Ultracut E ultramicrotome (Leica, Wetzlar, Germany). The 70–90 nm ultrathin sections were contrasted with uranyl acetate and lead citrate according to McDonald (1984) methodology and examined with the Zeiss EM 900 transmission electron microscope at 80 kV (TEM). To determine pigment composition in erythrophores, unstained skin samples were placed in Ringer's solution, 30% NH_4_OH due to dissolve pteridines or in 100% acetone due to dissolve carotenoids (Junqueira et al. 1978; Saenko et al. 2013; Wijnen et al. 2007), After that samples were observed and photographed under stereomicroscope Zeiss Stemi SV11 (Carl Zeiss, Oberkochen, Germany) with AxioCam ERc5s camera.

## Results

**Fig. 2:**
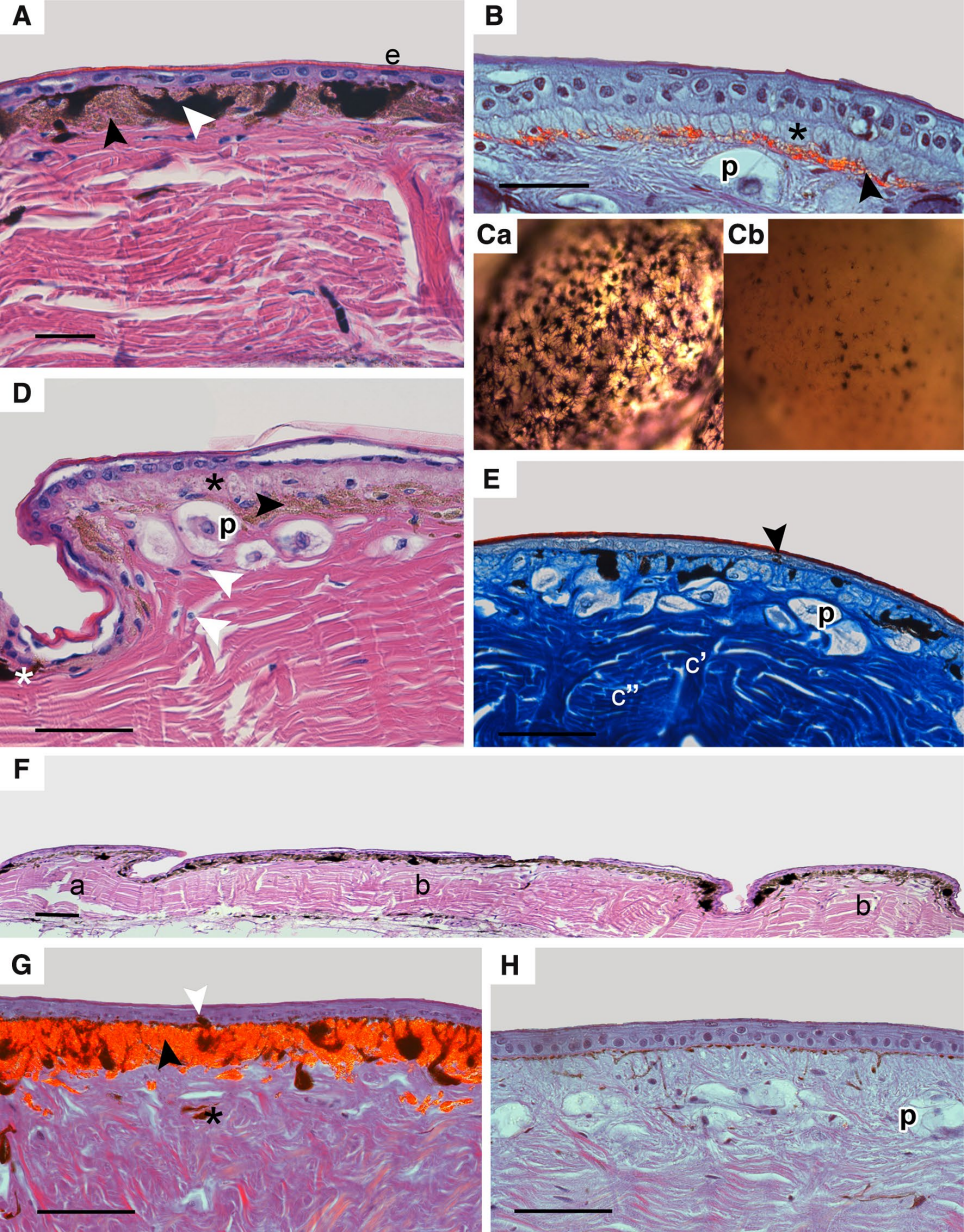
Distribution of chromatophores in tokay gecko skin; *scalebar* 50 µm. **A, D**, and **F**—H&E, **Ca, Cb**—micrograph of the skin *in toto* in transmitted light, **E**—Mallory’s stain, **B**, **G** and **H**—DIC (Nomarski contrast). **A** Blue pigmented area from dorsal part of body; epidermis (***e***), single melanophores with processes (*white arrowhead*), numerous iridophores (*black arrowhead*). **B** Orange/red pigmented area from dorsal part of head with clearly visible layer of iridophores (*black arrowhead*) and erythrophores (*black asterisk*). Parenchymatous cells (***p***) are visible in dermis under chromatophores. ***Ca, b***. Micrographs of the skin in toto in transmitted light from blue area (***Ca***), with high number of melanophores with melanin-filled processes and from orange/red area (***Cb***) where melanophores are visible in smaller numbers with melanosomes in perinuclear part of these cells (***Cb***). **D** Orange/red pigmented area from back where erythrophores are more numerous (*black asterisk*) than in blue pigmented area, a single layer of iridophores (*black arrowhead*), isolated melanophores (*white asterisk*), fibrocytes and fibroblasts visible in dermis (*white arrowhead*). Parenchymatous cells (***p***) are visible in dermis under chromatophores. **E**. Orange/red area from back with single melanocyte in epidermis (*black arrowhead*), characteristic of tuberculate scales bundles of collagen fibres with different orientation (***c’***, ***c”***), and parenchymatous cells (***p***) under chromatophores. **F** Two types of scales from dorsal part of back: overlapping scale (***a***) and tuberculate scales (***b***). **G** Blue area from dorsal part of tail with layer of iridophores (*black arrowhead*). Isolated melanocyte in epidermis (*white arrowhead*) and frequently deeper located melanophores (*black asterisk*). **H** Orange/red area from ventral part of trunk without iridophores (lack of contrasted cells). Parenchymatous cells (***p***) are visible under chromatophores

In this study, tokay gecko skin was composed of two typical layers as in other reptiles—an outer epidermis and an inner dermis. Skin samples analysed in H&E and Mallory’s stain were identified as stage one during the sloughing cycle according to Maderson (1966). The epidermal layer consisted of a stratum germinativum and a keratinized layer (stratum corneum) (Fig. 2A, D). The stratum germinativum was composed of 1 to 2 layers of epithelial cells above the basement membrane (Fig. 2A–E). The dermis contains chromatophores, connective tissue cells as fibroblasts and fibrocytes, and collagen fibres in the extracellular matrix (Fig. 2E). The bundles of collagen fibres have different orientation (Fig. 2E). Two different types of scales were identified: tuberculate scales and between them overlapping scales with different collagen fibres arrangement. Presence of numerous vertical bundles of collagen fibres were characteristic of tuberculate scales. Scales were separated by a hinge region where chromatophores were less numerous, epithelial cells were flattened, and thin stratum corneum was less pronounced (Fig. 2F).

**Fig. 3:**
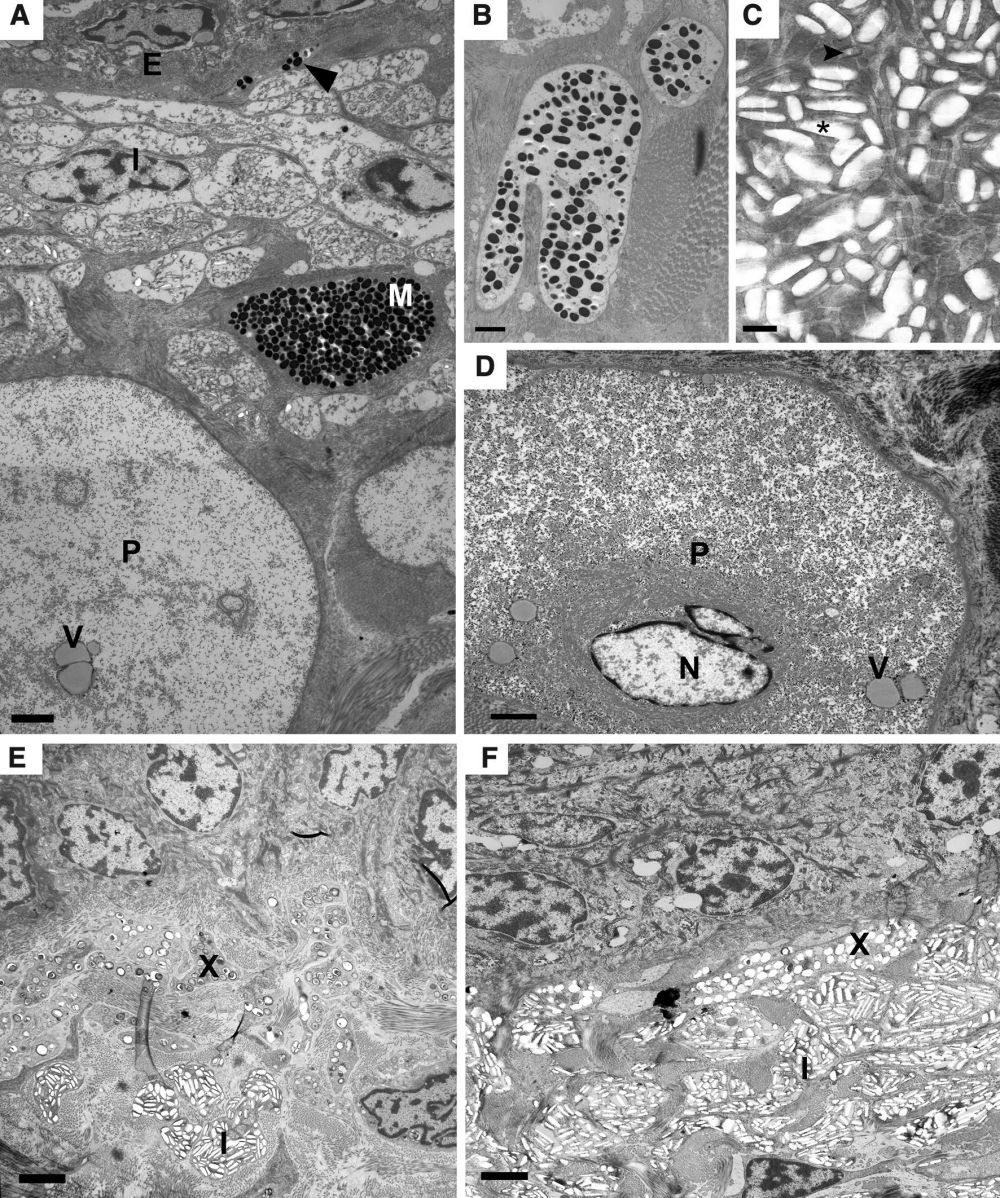
Ultrastructure of dermal pigment cells in tokay gecko skin. **A** Panorama of the epidermis with epithelial cells (***E***) and dermis with iridophores (***I***) located under basement membrane, and deeper located dermal melanophore (***M***) and parenchymatous cell (***P***) with vesicles (***V***); between iridophores and basement membrane melanophore processes (*black arrowhead*) are noted, *scalebar* 2.5 µm. **B** Melanophore processes from the deeper part of the dermis with smaller amount of melanosomes, *scalebar* 1.7 µm. **C** Rectangular shaped intact guanine crystals in iridophore cytoplasm (*black arrowhead*) and holes where crystal were lost (*black asterisk*), *scalebar* 0.4 µm. **D** Parenchymatous cell (***P***) with visible nucleus (***N***) and vesicles (***V***), *scalebar* 2.5 µm. **E** Numerous erythrophores (***X***) and single iridophores (***I***) observed in orange/red pigmented skin, *scalebar* 2.5 µm. **F** Single erythrophores (***X***) noted in blue pigmented area where iridophores (***I***) are more numerous, *scalebar* 2.7 µm

Melanophores were present in both blue and orange/red areas of the dorsal and ventral part of the head, body, and tail but in different numbers, as observed in sections stained with H&E, Mallory’s stain, in Nomarski contrast and in transmitted light (Fig. 2A–D, G, H). Isolated epidermal melanocytes were found both in blue and orange/red areas (Fig. 2E, G). Bodies and processes of dermal melanophores were arranged in a single layer under the basement membrane. However, isolated melanophores were also found in the deeper part of the dermis (Fig. 2G). Melanophores visible in the orange/red area had a smaller number of processes than in the blue regions of the skin (Fig. 2C). Analysis of light microscopy sections confirmed the presence of erythrophores and iridophores in all regions studied. Positive test with 30% NH_4_OH where pteridines were removed and negative test with 100% acetone, suggest that erythrophores contained pteridines in pterynosomes (Fig. 1C–E). Erythrophores were the superficial layer of chromatophores (Fig. 2B, D, H) when were visible in light microscopy slides. These cells were present both in blue and orange/red areas but in different numbers. Erythrophores were absent or in small number in blue regions (Figs. 2A, G, 3F) and definitely more numerous in orange/red areas (Figs. 1F, 2B, D). In H&E staining melanophores and iridophores were observed as a single layer of alternately arranged cells, which were located just under the basement membrane (Fig. 2A, G) in blue regions where erythrophores were absent or iridophores were found under erythrophores in orange/red regions (Fig. 2B). However, the TEM samples revealed that iridophores were the most superficial layer of dermal chromatophores that dominate in blue areas both in the dorsal and ventral parts of the head, body, and tail (Fig. 3A, F), while in orange/red areas they were less numerous or even absent and were located under erythrophores (Figs. 2B, D, 3E). Parenchymatous cells were observed in dermis under chromatophores (Fig. 2B, D, E, H).

TEM observation of melanophores showed typical electron-dense melanin containing granules—melanosomes (Fig. 3A, B). Superficially located melanophores contained large number of melanosomes in contrast to those found in the deeper region of the skin (Fig. 3B). Melanophore processes can be observed at a large distance from the cell body, which suggests the ability to penetrate between other chromatophores (Fig. 3A). Iridophores in TEM observation contained rectangular shaped, similar-sized guanine crystals that were evenly arranged around the cell nucleus (Fig. 3A, C, E, F). Intact guanine crystals and post guanine crystals holes were disorganized. During TEM observation we also found large non pigmentary cells located under chromatophores in dermis (Fig. 3A, D).

## Discussion

The presented study revealed three types of chromatophores, including melanophores, erythrophores, and iridophores in natural colouration of the *G. gecko* skin. The arrangement of these cells does not create a functional “chromatophore unit” like in some other reptiles or amphibians (Bagnara et al. 1968; Bagnara 1983; Taylor and Hadley 1970; Taylor and Bagnara 1972). In this study, melanophores were characterised by a large cell body and melanosome-containing processes like in other squamates as *A. carolinensis, P. latiscutatus*, and *S. punctaus* (Alexander and Farenbach 1969; Kuriyama et al. 2006; Alibardi 2012; Saenko et al. 2013). However, melanophores lying deeper in the dermis had larger and less numerous melanosomes in the processes. Melanophores had different number of processes between blue and orange/red areas of the skin. Melanophores with numerous processes were typical for blue areas while those with few processes were usually found in orange/red areas. The existence of melanophores in blue pigmented skin was also described in *P. latiscutatus* (Kuriyama et al. 2006). Xanthophores or erythrophores are usually filled with pteridines or carotenoid granules in other squamates (Bagnara 1966). In tokay gecko, erythrophores containing pteridines were more numerous in orange/red areas but absent or only in small numbers in blue areas. They were the superficial located chromatophores lying above the iridophores and melanophores. In blue areas deprived of erythrophores iridophores were the most superficial layer of chromatophores, but in orange/red areas of the skin they were found under erythrophores. In reptiles iridophores participate in skin colouration and in thermoregulation (Bagnara 1966; Sherbrooke and Frost 1989; Bagnara et al. 2007; Saenko et al. 2013; Teyssier et al. 2015). In viper of the genus *Bothrops*, they generate a green structural colouration reflecting the light through the outermost located xanthophores (Gosner 1989). The presence of iridophores observed under the light microscope is associated with a dominant green–blue colour, but when they are accompanied by xanthophores a green-yellow colour occurs (Rohrlich and Porter 1972). However, recent studies on panther chameleon revealed that iridophores are responsible for colour change in chameleons (Teyssier et al. 2015). In our study, orange/red areas of the skin harboured less iridophores than the blue ones. Additionally, large cells described as parenchymatous cells (Bauer et al. 1989) were noted in dermis just under chromatophores layer. In cited study prenchymatous cells take part in weaken physical skin strength.

*G. gecko* is a species from the genus *Gekko*, order Gekkonidae, and Gekkota genera (Pyron et al. 2013; Gamble et al. 2015). Chromatophores from Phelsuma genus, Gekkonidae order, Gekkota genera were described for diurnal species, including species *P. klemmeri, P. quadriocellata, P. lineata, P. laticauda*, and *P. grandis* and nocturnal species *Eublepharis macularius* from Eublepharidae order and Gekkota genera (Saenko et al. 2013; Gamble et al. 2015; Szydłowski et al. 2016). Iridophores that are present in the skin of species from Phelsuma genus participate in green skin colouration and day activity, but in nocturnal species—*E. macularius* are absent. According to the recent study on the evolution of diurnality in geckos, *G. gecko* and *E. macularius* are nocturnal species (Gamble et al. 2015) but, as the present study revealed, only *G. gecko* has iridophores. In Gekkonidae both diurnal and nocturnal activity exist. Primal activity common for Eublepharidae and Gekkonidae is nocturnality (Gamble et al. 2015) which suggest that iridophores in tokay gecko can be a feature inherited from ancestors due to other phylogeny and adaptation to different environments. This environmental adaptation in blue and orange/red colouration in tokay gecko should be discussed. Although natural skin colouration of tokay gecko is not cryptic (for human eye colour perception) in a natural environment, it could be part of a strategy to avoid being noticed by predators with different colour vision. Similar mechanism is observed in blue-yellow reef fish, whose colouration merges into the environmental background when observed from a longer distance (Marshall 2000). Studies on different colour vision by predators suggest another type of explanation for bright skin pigmentation of tokay gecko, which could be less conspicuous for predators (Hastad et al. 2005). Even if tokay gecko could be visible for predators, colourful and bright skin pigmentation might be the “toxicity strategy” as warning signal for predators like kind of mimicry (Darst et al. 2006).

In summary, our study explains the relation between number and composition of chromatophores in *G. gecko* skin pigmentation. In each skin colouration all three types of chromatophores could be present but in different numbers. However, orange/red pigmented areas demonstrate the predominant number of erythrophores, smaller number of melanophores with processes, and the smallest number of iridophores. In areas where all types of chromatophores are present, superficial iridophores, deeper located melanophores, and erythrophores were observed.
